# Reconstructing coral calcification fluid dissolved inorganic carbon chemistry from skeletal boron: An exploration of potential controls on coral aragonite B/Ca

**DOI:** 10.1016/j.heliyon.2017.e00387

**Published:** 2017-08-30

**Authors:** Nicola Allison

**Affiliations:** School of Earth and Environmental Sciences, University of St Andrews, Irvine Building, North Street, St Andrews, Fife, KY16 9AL, UK

**Keywords:** Geochemistry, Geology, Oceanography

## Abstract

The boron geochemistry of coral skeletons reflects the dissolved inorganic carbon (DIC) chemistry of the calcification fluid from which the skeletons precipitates and may be a valuable tool to investigate the effects of climate change on coral calcification. In this paper I calculate the predicted B/Ca of aragonite precipitating from seawater based fluids as a function of pH, [DIC] and [Ca^2+^]. I consider how different co-precipitating DIC species affect aragonite B/Ca and also estimate the impact of variations in the B(OH)_4_^−^/co-precipitating DIC aragonite partition coefficient (K_D_), which may be associated with changes in the DIC and Ca^2+^ chemistry of the calcification fluid. The coral skeletal B/Ca versus calcification fluid pH relationships reported previously can be reproduced by estimating B(OH)_4_^−^ and co-precipitating DIC speciation as a function of pH_CF_ and assuming that K_D_ are constant i.e. unaffected by calcification fluid saturation state. Assuming that B(OH)_4_^−^ co-precipitates with CO_3_^2−^, then observed patterns can be reproduced by a fluid with approximately constant [DIC] i.e. increasing pH_CF_ concentrates CO_3_^2−^, as a function of DIC speciation. Assuming that B(OH)_4_^−^ co-precipitates with HCO_3_^−^ only or CO_3_^2−^ + HCO_3_^−^ then the observed patterns can be reproduced if [DIC]_CF_ and pH_CF_ are positively related i.e. if DIC is increasingly concentrated in the calcification fluid at higher pH_CF_ probably by CO_2_ diffusion into the calcification site.

## 1 Introduction

The boron geochemistry of coral skeletons offers a potential method to reconstruct the dissolved inorganic carbon (DIC) chemistry of the coral calcification fluid (Allison et al., 2014) and to determine how it responds to environmental change. Coral biomineralisation underpins the production of the coral reef structure and understanding the controls on the calcification process is key to predicting the impacts of increasing seawater temperatures (Hoegh-Guldberg et al., 2007) and pCO_2_ (ocean acidification, Caldeira and Wickett, 2003) on reef development.

Dissolved boron in seawater occurs primarily as boric acid, B(OH)_3_, and borate, B(OH)_4_^−^, and speciation is controlled by ambient pH (Hemming and Hanson, 1992). Most reports suggest that B(OH)_4_^−^ is selectively incorporated into aragonite (Sen et al., 1994; Noireaux et al., 2015) substituting for CO_3_^2−^ in the lattice (Balan et al., 2016) and is depleted in ^11^B compared to B(OH)_3_ (Klochko et al., 2006). Hence aragonite δ^11^B reflects the pH of the fluid from which it precipitates (Allison et al., 2010) while [B] (usually measured as B/Ca) reflects both fluid pH and the concentration of the DIC species competing with B(OH)_4_^−^ for inclusion in the carbonate (Allen et al., 2012).

Coral aragonite precipitates from an extracellular calcifying fluid enclosed in a semi-isolated space between the coral tissue and underlying skeleton (Clode and Marshall, 2002). The calcification fluid is derived from seawater (Tambutté et al., 2012) but corals actively increase its pH above that of seawater (Al-Horani et al., 2003; Venn et al., 2011; Venn et al., 2012) altering the fluid DIC chemistry. To date there has been no direct comparison of coral skeletal δ^11^B and calcifying fluid pH to confirm that skeletal δ^11^B records actual calcifying fluid pH. Fluid pH can be estimated by direct observation of pH sensitive dyes at the calcification site, The mean pH of dye-based observations in the light and dark of the branching coral *Stylophora pistillata* (Venn et al., 2011) is in good agreement with fluid pH estimates derived from skeletal δ^11^B of different individuals of the same coral species cultured at present day seawater pCO_2_ (Krief et al., 2010), when corrected for the typical ratio of light:dark calcification rates (Gattuso et al., 1999). Positive trends are observed between seawater pH and calcification fluid pH when fluid pH is either inferred from skeletal δ^11^B (Honisch et al., 2004; Reynaud et al., 2004) or directly measured (Venn et al., 2012). These collective observations suggest that skeletal δ^11^B reflects calcification fluid pH changes.

Increasing calcification fluid pH shifts the fluid DIC equilibrium in favour of carbonate (CO_3_^2−^) at the expense of CO_2_ and bicarbonate (HCO_3_^−^) and creates a concentration gradient facilitating the diffusion of CO_2_ from the overlying coral tissue into the fluid (Erez, 1978). This CO_2_ can react to form more HCO_3_^−^ and CO_3_^2−^, thereby increasing calcification fluid DIC. Preliminary application of the skeletal B/Ca − calcification fluid DIC proxy suggested that [DIC] at the coral calcification site is increased above that of seawater and that bicarbonate contributes to the DIC pool used for calcification (Allison et al., 2014). The B(OH)_4_^−^:aragonite partition coefficient utilized in this study was estimated from the B/Ca analysis of a secondary aragonite cement in a fossil coral coupled with alkalinity measurements of pore fluid and δ^11^B of the cement (indicating pore fluid pH). Recent inorganic aragonite precipitation studies indicate that the borate:aragonite partition coefficient can be highly variable (Mavromatis et al., 2015; Holcomb et al., 2016) and is probably affected by the saturation state of the precipitating fluid (Holcomb et al., 2016). This complicates the interpretation of coral skeletal [B].

**Fig. 1:**
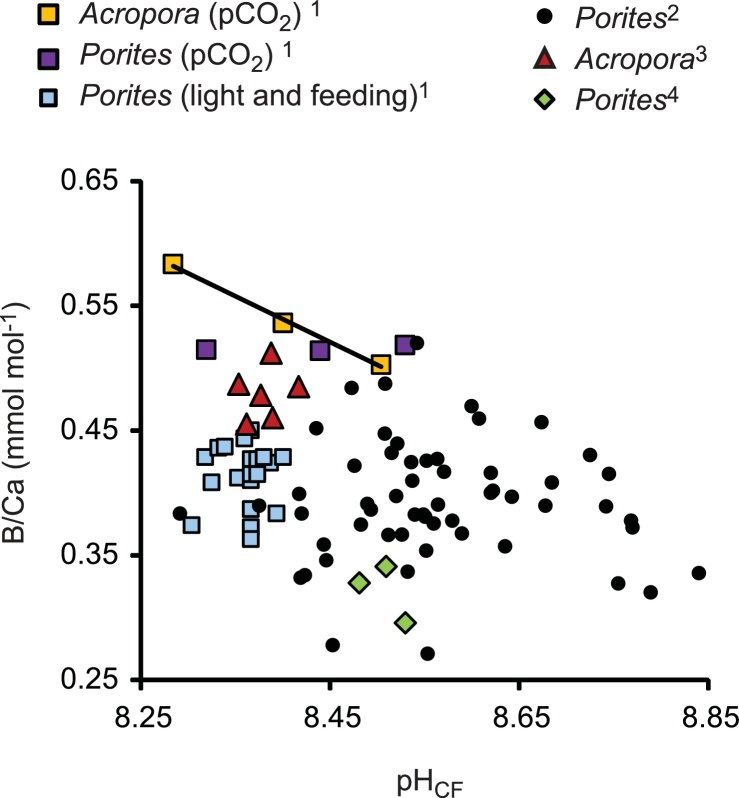
Published relationships between coral calcification pH_CF_ (inferred from δ^11^B) and skeletal B/Ca. Data sources are: 1 = Honisch et al. (2004); 2 = Allison et al. (2010), 3 = Dissard et al. (2012) and 4 = Allison et al. (2014). All data are corrected to the pH total scale. None of the datasets exhibit significant correlations between pH_CF_ and B/Ca with the exception of *Acropora* sp. corals cultured over a range of seawater pCO_2_ (Honisch et al., 2004).

In this paper I explore how variations in calcification fluid DIC, [Ca^2+^] and pH affect skeletal B/Ca. Both boron and DIC speciation are pH dependent while variations in fluid DIC affect the saturation state of the fluid. I calculate the predicted B/Ca of aragonite precipitating from seawater based fluids with DIC, B and Ca^2+^ chemistries which are comparable to those of coral calcification fluids. Most coral datasets do not exhibit significant correlations between calcification fluid pH (inferred from δ^11^B) and skeletal B/Ca (Fig. 1) and it is timely to consider why this is. It is unclear which dissolved inorganic carbon (DIC) species is/are involved in aragonite precipitation. CO_3_^2−^ is predominantly incorporated in the crystal lattice (Von Euw et al., 2017) but HCO_3_^−^ may also be involved in mineral precipitation (Wolthers et al., 2012; Mass et al., 2013; Von Euw et al., 2017). I consider how different co-precipitating DIC species affect aragonite B/Ca and also estimate the impact of variations in the B(OH)_4_^−^/co-precipitating DIC aragonite partition coefficient, which may be associated with changes in the DIC and Ca^2+^ chemistry of the calcification fluid.

## 2 Methods

### 2.1 Impacts of coral processes on calcification fluid DIC chemistry

**Fig. 2:**
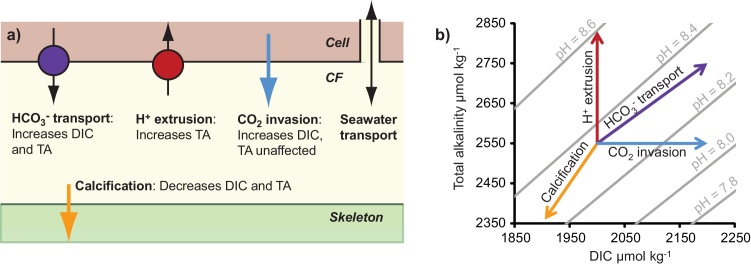
(a) The key processes affecting coral calcification fluid DIC chemistry and (b) their effects on seawater DIC, total alkalinity and pH_CF_.

The key processes affecting calcification fluid DIC chemistry and their effects are summarized in Fig. 2. All pH values are reported on the total scale and the subscript _CF_ denotes the DIC characteristics of the calcification fluid. Corals increase pH_CF_ above that of seawater e.g. using the antiporter Ca-ATPase (Ip et al., 1991; Marshall, 1996) which pumps Ca^2+^ into the calcification site in exchange for 2H^+^. H^+^ extrusion increases the total alkalinity of the fluid but does not affect DIC. Total alkalinity is defined as the number of moles of hydrogen equivalent to the excess of proton acceptors over proton donors in the fluid (Zeebe and Wolf-Gladrow, 2001) so proton extrusion increases total alkalinity and decreases [H^+^] in a 1:1 mol ratio. Increasing pH_CF_ favours the reaction of CO_2_ and H_2_O to form HCO_3_^−^ (and H^+^) and facilitates the diffusion of CO_2_ into the calcification site. This increases fluid [DIC] but does not affect total alkalinity. Bicarbonate anion transporters (BATs) convey HCO_3_^−^ into the calcification fluid, likely in exchange for Cl^−^ (Zoccola et al., 2015). This increases total alkalinity and DIC in a 1:1 mol ratio and ultimately decreases pH_CF_. Aragonite precipitation removes DIC and total alkalinity from the calcification fluid in a 1:2 mole ratio (one mole of the CO_3_^2−^ ion ultimately incorporated in the aragonite contains one mole of carbon but is doubly charged so contributes 2 moles to total alkalinity) and also decreases pH_CF_.

### 2.2 Calcification fluid chemistry and precipitation scenarios

I calculate the DIC chemistry of a seawater based calcification fluid and the B/Ca of aragonite precipitated from it under a range of different scenarios (summarised in Table 1) as a function of pH_CF_. The details of the scenarios are as follows:

**Table 1:** Summary of scenarios used to estimate coral calcification fluid chemistry and aragonite B/Ca.

Scenario	Detail of different scenarios
1. Co-precipitating DIC species	a) B(OH)_4_^−^ co-precipitates with CO_3_^2−^ b) B(OH)_4_^−^ co-precipitates with HCO_3_^−^ c) B(OH)_4_^−^ co-precipitates with CO_3_^2−^ + HCO_3_^−^
2. DIC_CF_	a) DIC_CF_ equals that of seawater (low DIC) b) DIC_CF_ is higher than that of seawater (high DIC)
3. Seawater pCO_2_	a) Seawater DIC is in equilibrium with 400 μatm CO_2_ b) Seawater DIC is in equilibrium with 800 μatm CO_2_
4. B(OH)_4_^−^/co-precipitating DIC K_D_	a) variable b) constant
5. [Ca^2+^]_CF_	a) [Ca^2+^]_CF_ covaries with proton extrusion b) [Ca^2+^]_CF_ is unaffected by proton extrusion

#### 2.2.1 Co-precipitating DIC species

I assume that either CO_3_^2−^ only, HCO_3_^−^ only, or both CO_3_^2−^ and HCO_3_^−^ co-precipitate with B(OH)_4_^−^ in the aragonite (Table 1, scenario 1).

#### 2.2.2 DIC_CF_ and seawater pCO_2_

I assume that the [DIC]_CF_ is either low or high and that the fluid is overlain or surrounded by coral tissues and/or ambient seawater in equilibrium with either 400 μatm CO_2_ or 800 μatm CO_2_. I assume that [DIC]_CF_ is either equivalent to that of seawater (the low DIC_CF_ scenario) or is higher than this (the high DIC_CF_ scenario), Table 1. In the case that [DIC]_CF_ is equivalent to that of seawater, I effectively calculate the DIC of the calcification fluid as a closed system. I do not infer that the fluid is closed to CO_2_ diffusion but rather that additions of CO_2_ to the calcification fluid by diffusion are balanced by loss of DIC to precipitation. This is supported by direct measurements of pH_CF_ and [CO_3_^2−^]_CF_ which indicate that DIC_CF_ is approximately constant and equal to that of ambient seawater (Cai et al., 2016). I assume that ambient seawater is in equilibrium with either 400 μatm CO_2_ (ambient seawater has pH 8 and [DIC] = 1796 μmol kg^‐1^) or 800 μatm CO_2_ (ambient seawater has pH 7.74 and [DIC] = 1911 μmol kg^‐1^). This doubling of seawater pCO_2_ indicates how DIC_CF_ is affected if the [CO_2_] of the coral tissue and body compartments is higher than that of ambient seawater (Cai et al., 2016). In the high DIC scenario, I assume that diffusion of CO_2_ into the calcification fluid increases [DIC]_CF_ approximately twofold above that of seawater at typical coral calcification fluid pH and at a seawater pCO_2_ of 400 μatm CO_2_. The transport rate of CO_2_ across a membrane can be expressed by Fick’s first law of diffusion as:

(1) Flux = −P. A. ΔC_w_

**Fig. 3:**
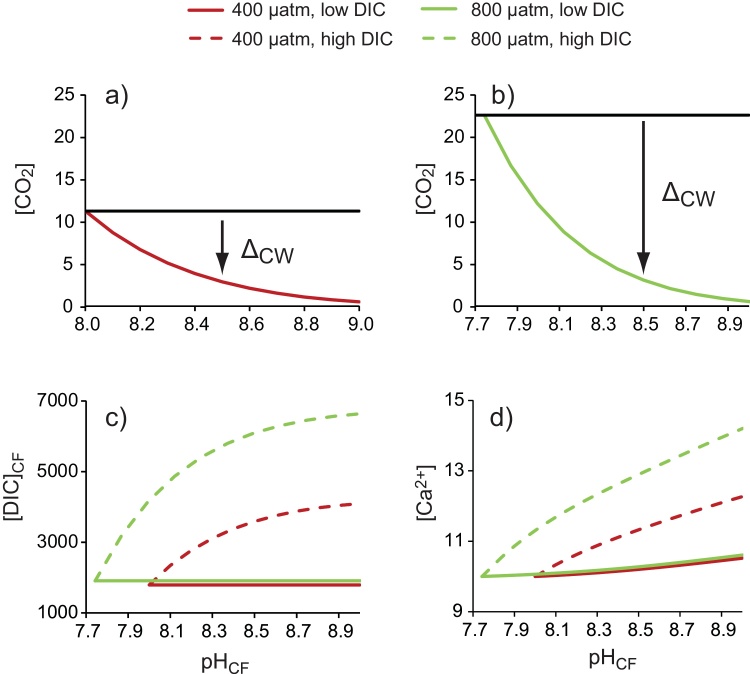
Estimated changes in fluid DIC (μmol kg^-1^) and [Ca^2+^] (mmol kg^-1^) as a function of pH_CF_. (a) and (b) [CO_2_] of a fluid with total [DIC] = 1796 μmol kg^−1^ (the typical [DIC] in seawater at equilibrium with 400 μatm) and 1911 μmol kg^−1^ (the typical [DIC] in seawater at equilibrium with 800 μatm CO_2_) respectively. Black horizontal lines indicate the [CO_2_] of an overlying ambient seawater at a) 400 μatm CO_2_ and b) 800 μatm CO_2_. These lines are extended across the entire pH_CF_ range to ease visualisation of ΔC_W_ although the pH of the overlying seawater is constant. ΔC_W_ indicates the CO_2_ concentration gradient between the calcification fluid and ambient seawater at pH 8.5 which facilitates CO_2_ diffusion from seawater into the fluid. (c) Total fluid [DIC] after any CO_2_ diffusion (calculated by assuming CO_2_ diffusion doubles fluid [DIC] at pH 8.5 and 400 μatm CO_2_ and scaling all other CO_2_ additions by diffusion as a linear function of ΔC_W_ at this pH. d) Fluid [Ca^2+^] calculated from fluid total alkalinity assuming that all proton extrusion is mediated by Ca-ATPase.

Where P = the membrane permeability, A = the diffusional area and ΔC_w_ the difference of CO_2_ concentrations in the water phase immediately adjacent to the two sides of the membrane (Endeward et al., 2014). Assuming that P and A remain constant, the flux of CO_2_ into the calcification fluid is controlled by the CO_2_ concentration difference between the calcification fluid and the overlying coral tissue. I estimate this concentration difference assuming that the [CO_2_] of the overlying tissue is the same as ambient seawater (11.3 μmol kg^−1^ and 22.6 μmol kg^−1^ in the 400 and 800 μatm CO_2_ scenarios respectively) and that the [CO_2_] of the calcification fluid reflects that of ambient seawater brought to pH_CF_ (Fig. 3a,b). I arbitrarily assume that CO_2_ diffusion doubles the [DIC]_CF_ at pH 8.5 (the typical mean coral calcification fluid pH, Allison et al., 2014) in the 400 μatm CO_2_ scenario (i.e. adding 1796 μmol kg^−1^ to the DIC_CF_) and scale the addition of DIC to the calcification fluid over the full pH range at 400 and 800 μatm CO_2_ as a linear function of ΔC_w_. This calculation is shown in detail in Appendix 1. I assume that additional H^+^ extrusion compensates for the pH decrease associated with the ingress of CO_2_ so that pH_CF_ is unaffected by this CO_2_ invasion. I calculate the final [DIC]_CF_ as the [DIC]_seawater_ plus CO_2_ added by diffusion (Fig. 3c).

I estimate the concentrations of DIC species and total alkalinity in the calcification fluid by setting pH_CF_ and [DIC]_CF_ and calculating all other carbonate system parameters with CO2sys version 2.0 (Pierrot et al., 2006) using the equilibrium constants for carbonic acid from Mehrbach et al. (1973), refit by Dickson and Millero (1987) and for KHSO_4_ from Dickson (1990). Total fluid boron was set to 416 μmol kg^−1^ (Uppstrom, 1974). I assume constant temperature (25 °C) and salinity (35).

#### 2.2.3 B(OH)_4_^−^/co-precipitating DIC aragonite K_D_

**Fig. 4:**
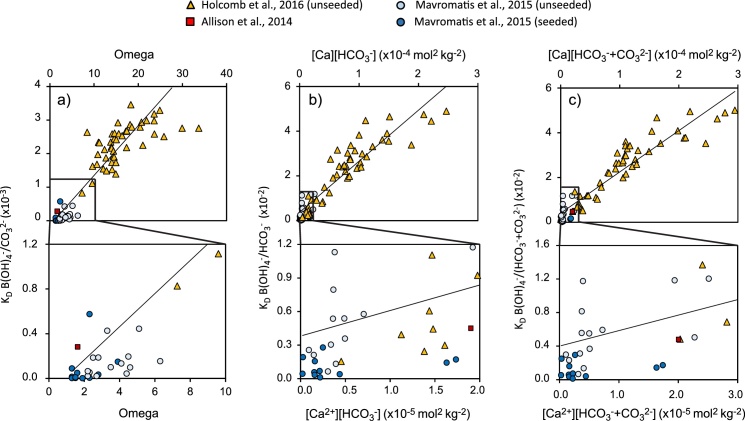
B(OH)_4_^−^/co-precipitating DIC aragonite partition coefficients (calculated from Mavromatis et al., 2015 and Holcomb et al., 2016) assuming that B(OH)_4_^−^ co-precipitates with (a) CO_3_^2−^ only, (b) HCO_3_^−^ only and (c) CO_3_^2−^ + HCO_3_^−^, as a function of the saturation of the precipitating fluid defined as (a) Ω_aragonite_, (b) [Ca^2+^][HCO_3_^−^] and c) [Ca^2+^][CO_3_^2−^ + HCO_3_^−^], all concentrations in mol kg^-1^. The lower half of each figure is an expanded view of the boxed section in the upper figure. The partition coefficients determined from analysis of an inorganic cement in a fossil coral (Allison et al., 2014) are also shown.

To estimate the B(OH)_4_^−^/co-precipitating DIC aragonite partition coefficients (hereafter abbreviated to K_D_) to apply in the calculations I combine data from two studies that measured boron partitioning in inorganic aragonite at ∼25 °C (Mavromatis et al., 2015; Holcomb et al., 2016). I exclude data collected from experiments conducted in the presence of buffers. I calculate the B(OH)_4_^−^/co-precipitating DIC ratios of the precipitation fluids and combine these with the B/Ca of the precipitated aragonites to estimate K_D_. Aragonite precipitation rate is typically positively correlated with the precipitation fluid saturation (Burton and Walter, 1987), an indication of the concentrations of solute ions in solution, and trace element partitioning may be affected by mineral precipitation rate (Watson, 1994; Elderfield et al., 1996). I plot the K_D_ against the saturation of the precipitating fluid defined as Ω_aragonite_ ([Ca^2+^][CO_3_^2−^]/K*sp_aragonite_) for scenario 1a, [Ca^2+^][HCO_3_^−^] for scenario 1b and [Ca^2+^][HCO_3_^−^ + CO_3_^2−^] for scenario 1c. The experiments of Mavromatis et al. (2015) and Holcomb et al. (2016) were conducted in 0.1–0.2 M NaCl solutions and seawater respectively. I have not corrected solute concentrations to activities. I have used stoichiometric equilibrium constants (conventionally denoted as K*), which correct for ionic strength and utilize ion concentrations rather than activities, for all calculations. There are no obvious offsets between the data of Holcomb et al. (2016) and Mavromatis et al. (2015) and all 3 partition coefficients are linearly positively correlated with fluid saturation (Fig. 4) according to the relationships:

(2) K_D_ B(OH)_4_^−^/CO_3_^2−^ = 1.48 × 10^−4^omega − 1.30 × 10^−4^ (r^2^ = 0.86)

(3) K_D_ B(OH)_4_^−^/HCO_3_^−^ = 1.85 × 10^2^[Ca^2+^][HCO_3_^−^] + 4.01 × 10^−3^ (r^2^ = 0.89)

(4) K_D_ B(OH)_4_^−^/(CO_3_^2−^ + HCO_3_^−^) = 2.28 × 10^2^[Ca^2+^][CO_3_^2−^ + HCO_3_^−^] + 3.81 × 10^−3^ (r^2^ = 0.87)

The incorporation of boron in aragonite is not fully understood but this observation is consistent with the growth entrapment model (e.g. Watson, 1994) which suggests that trace elements are captured as impurities at the crystal surface before they can diffuse back into the surrounding fluid. B/Ca incorporation in calcite is positively correlated with crystal extension (Gabitov et al., 2014) and precipitation rate (Uchikawa et al., 2015). K_D_ B(OH)_4_^−^/CO_3_^2−^ is also positively correlated with precipitation rate in aragonite but K_D_ B(OH)_4_^−^/HCO_3_^−^ and K_D_ B(OH)_4_^−^/(CO_3_^2−^ + HCO_3_^−^) do not show this relationship (Holcomb et al., 2016), although precipitation rate is not necessarily a good indication of crystal extension rate. In the growth entrapment model, maximum entrapment is reached at high crystal growth rates and K_D_ becomes independent of crystal growth rate. This may just be occurring at the highest fluid saturation states in Fig. 4.

In calculating aragonite B/Ca I assume that K_D_ are either dependent on fluid saturation state or are constant (scenarios 4a and b, Table 1). In the first case I am inferring that higher fluid saturation stimulates high crystal extension rates leading to relatively high B incorporation. In the second case I am assuming that high fluid saturation does not affect crystal extension rate. Coral calcification rates are positively correlated with the saturation states of the calcification fluid (Allison et al., 2014) and seawater (Gattuso et al., 1998). However it does not automatically follow that the growth rates of coral skeletal crystals increase at high saturation states. In the massive *Porites* spp. corals, typically used for palaeoenvironmental reconstruction, linear extension of the skeleton occurs by the deposition of centres of calcification or centres of rapid accretion (Nothdurft and Webb, 2007) which are aligned perpendicular to the plane of the skeleton surface. Fasciculi, composed of bundles of acicular aragonite crystals radiate out from these centres and are aligned perpendicular to the centres and approximately parallel to the skeleton surface. These make up the bulk of the skeleton (Allison, 1996). Fast coral calcification rates could reflect rapid extension of fasciculi crystals but could equally well be explained by constant extension rates of a larger volume of fasciculi crystals.

**Fig. 5:**
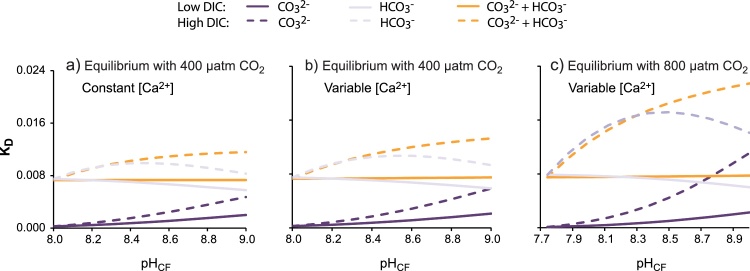
K_D_ as a function of pH_CF_ with (a) constant [Ca^2+^] and equilibrium with 400 μatm CO_2_, (b) variable [Ca^2+^] and equilibrium with 400 μatm CO_2_ and (c) variable [Ca^2+^] and equilibrium with 800 μatm CO_2_. Colours indicate different co-precipitating DIC species, solid lines indicate low DIC and dashed lines indicate high DIC.

In the case that B(OH)_4_^−^/co-precipitating DIC K_D_ is dependent on fluid saturation state (scenario 4a) I calculate calcification fluid saturation as [Ca^2+^]_CF_[CO_3_^2−^]_CF_/K*sp_aragonite_ for scenario 1a, [Ca^2+^]_CF_[HCO_3_^−^]_CF_ for scenario 1b and [Ca^2+^]_CF_[HCO_3_^−^ + CO_3_^2−^]_CF_ for scenario 1c and then calculate K_D_ from Eqs. (2)–(4) (Fig. 5). In the case that K_D_ is independent of fluid saturation, I calculate the saturation state for a typical coral fluid based on direct measurements of [CO_3_^2−^]_CF_ at pH_CF_ = 8.55 (pH = 8.7 NBS scale, Cai et al., 2016), broadly comparable to the mean pH_CF_ derived from δ^11^B of massive *Porites* spp. field corals (Allison et al., 2014). I calculate K_D_ of 0.00105, 0.00636 and 0.00669 for B(OH)_4_^−^/CO_3_^2−^, B(OH)_4_^−^/HCO_3_^−^ and B(OH)_4_^−^/(CO_3_^2−^ + HCO_3_^−^) respectively. I apply these same K_D_ to all scenarios in which I use a constant K_D_, irrespective of [co-precipitating DIC]_CF_, [Ca^2+^]_CF_ or CO_2_ atmosphere.

#### 2.2.4 [Ca^2+^]_CF_

[Ca^2+^]_CF_ affects the saturation state of the calcification fluid and subsequently may influence the B(OH)_4_^−^:aragonite partition coefficient (see section 2.2.3). I adopt 2 approaches to estimate [Ca^2+^]_CF_. In scenario 5a I assume that all proton extrusion from the calcification fluid is mediated by Ca-ATPase. This enzyme pumps 2H^+^ out of the fluid for every Ca^2+^ pumped in, thereby increasing fluid total alkalinity and [Ca^2+^] in a 2:1 mole ratio. I use the total alkalinity of the calcification fluid (calculated in the DIC_CF_ and seawater pCO_2_ section) to infer the activity of the enzyme and to calculate fluid [Ca^2+^] (Fig. 3d) assuming that [Ca^2+^] of the fluid at pH 8 is that of ambient seawater (10 mmol kg^‐1^). Aragonite precipitation removes total alkalinity and Ca^2+^ in a 2:1 mole ratio so does not influence this approach. In scenario 5b I assume that proton extrusion is mediated by an alternative ATPase which does not affect fluid [Ca^2+^]. Direct measurements of coral calcification fluids suggest that fluid [Ca^2+^] is similar to seawater (within 5%, Al-Horani et al., 2003) and in scenario 5b I assume a constant [Ca^2+^]_CF_ of 10 mmol kg^−1^.

#### 2.2.5 Calculating aragonite B/Ca

I calculate the B/Ca of aragonite precipitated from the calcification fluid under the various scenarios. Aragonite B/Ca equates to aragonite B(OH)_4_^−^/CO_3_^2−^ as Ca and C are equimolar in CaCO_3_. I assume that dissolved boron is transported into the calcification fluid in seawater and that the total [B] of the fluid is the same as seawater i.e. 416 μmol kg^−1^ (Uppstrom, 1974). I assume this concentration is constant for all scenarios as aragonite precipitation has little effect on [B]_CF_ due to the low B(OH)_4_^−^/co-precipitating DIC aragonite partition coefficients (Fig. 4). I estimate [B(OH)_4_^−^]_CF_ using K*_B_ = 2.527 × 10^−9^ (Dickson, 1990) and calculate fluid B(OH)_4_^−^/co-precipitating DIC. I multiply fluid B(OH)_4_^−^/co-precipitating DIC by K_D_ to calculate aragonite B/Ca.

## 3 Results and discussion

### 3.1 [DIC]_CF_

δ^11^B of *Porites* spp. field corals suggests that mean coral pH_CF_ is ∼8.5 (Allison et al., 2014). Here I assume that [DIC]_CF_ is either comparable to that of seawater or is doubled by CO_2_ diffusion at pH_CF_ = 8.5 and 400 μatm CO_2_. It is probable that these calculations reproduce the approximate DIC chemistry of the coral calcification fluid. Covariation of pH_CF_ and DIC_CF_ suggests that [DIC]_CF_ is broadly similar to that of seawater (Cai et al., 2016). While coral uptake of ^45^Ca and ^14^C in dual labelling experiments suggesting that the majority of skeletal carbon is derived from CO_2_ which diffuses into the calcification site rather than from seawater (Erez, 1978).

In scenarios where [DIC]_CF_ increases above that of seawater I describe the CO_2_ concentration gradient (Δ_CW_) between the coral tissue and the calcification fluid assuming that the [CO_2_] of the overlying tissue is the same as ambient seawater and that fluid [CO_2_] reflects that of ambient seawater brought to pH_CF_. The [CO_2_] of a fluid brought to pH_CF_ is exponentially related to pH_CF_ i.e. at high pH_CF_ fluid [CO_2_] becomes progressively smaller (Fig. 3a,b). Any increase in Δ_CWat_ high pH_CF_ is also progressively smaller and fluid [DIC] eventually begins to plateau. The final [DIC]_CF_ ranges from 1796 μmol kg^−1^ and 1911 μmol kg^−1^ (the low DIC scenarios) to ∼4100 μmol kg^−1^ and ∼6600 μmol kg^‐1^ at pH_CF_ 9 for the 400 and 800 μatm CO_2_ high DIC scenarios respectively (Fig. 3c). [DIC]_CF_ is higher at 800 μatm CO_2_ as the larger ΔC_W_ facilitates more CO_2_ diffusion into the calcification fluid.

### 3.2 [Ca^2+^]_CF_

I estimate the [Ca^2+^]_CF_ assuming that either Ca-ATPase is responsible for all proton extrusion (resulting in pumping of Ca^2+^ into the calcification site) or that other, non-Ca pumping, enzymes fulfil this role (resulting in no change to [Ca^2+^]_CF_). In the first scenario [Ca^2+^]_CF_ increases by 5% and 6% above that of seawater at 400 and 800 μatm CO_2_ respectively at low DIC and by 23% and 42% respectively at high DIC (Fig. 3d). The concentration increase is higher in the high DIC scenarios because increased Ca-ATPase activity is required to attain pH_CF_ when more CO_2_ diffuses into the calcification fluid. Similarly [Ca^2+^]_CF_ increases more at 800 μatm CO_2_ than at 400 μatm CO_2_ reflecting the higher Ca-ATPase activity to reach pH_CF_ starting from an ambient seawater with pH ∼7.7.

### 3.3 Fluid B(OH)_4_^−^/[co-precipitating DIC]

**Fig. 6:**
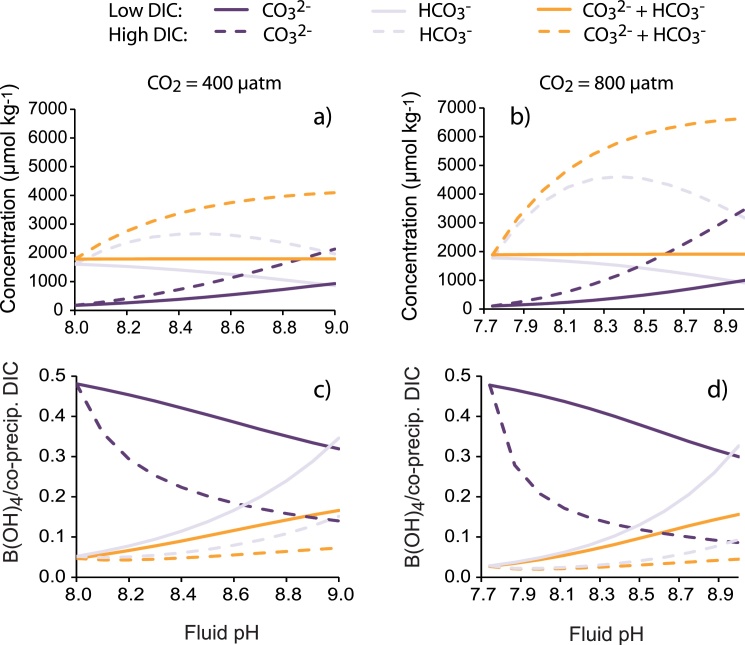
Estimated calcification fluid DIC chemistry (a and b) and fluid B(OH)_4_^−^/co-precipitating DIC (c and d) as a function of fluid pH. (a) and (c) indicate scenarios at 400 μatm CO_2_ and b) and d) indicate scenarios at 800 μatm CO_2_. Colours indicate different co-precipitating DIC species, solid lines indicate low DIC and dashed lines indicate high DIC.

Concentrations of individual co-precipitating DIC species under the different scenarios are illustrated in Fig. 6. In the low DIC scenarios I treat the calcification fluid as a closed system and the concentrations of co-precipitating DIC species are dependent on pH_CF_. [CO_3_^2−^]_CF_ is positively correlated with pH_CF_, [HCO_3_^−^]_CF_ is negatively correlated and [CO_3_^2−^ + HCO_3_^−^]_CF_ is almost constant (Fig. 6a,b solid lines). The high DIC scenarios (when CO_2_ invasion increases [DIC]_CF_ above that of seawater) are associated with higher [co-precipitating DIC]_CF_ than the low DIC at comparable pH_CF_. However while both [CO_3_^2−^]_CF_ and [CO_3_^2−^+ HCO_3_^−^]_CF_ are positively correlated with pH_CF_, [HCO_3_^−^]_CF_ increases to a maximum at ∼ pH 8.3–8.5 and then begins to decrease again (Fig. 5a,b dotted lines). Above this pH_CF_, any further increase in [DIC]_CF_ is relatively small (Fig. 3c) and the decrease in proportional abundance of HCO_3_^−^ as pH_CF_ increases outweighs any increase in total [DIC]_CF_.

**Fig. 7:**
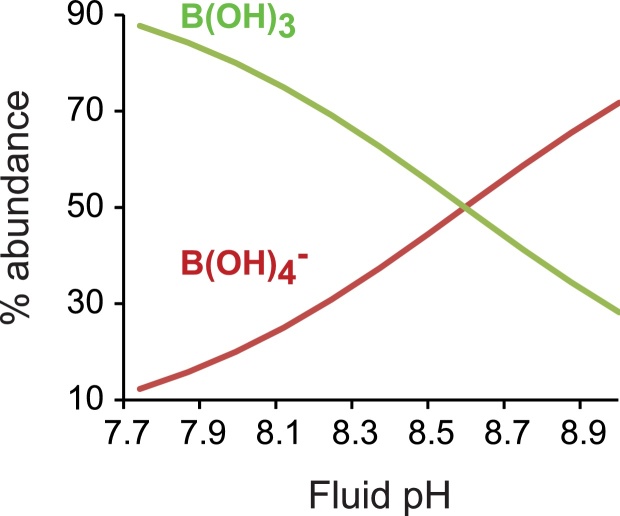
Percentage abundance of B(OH)_3_ and B(OH)_4_^−^ as a function of pH (T = 25 °C, S = 35).

B(OH)_3_ and B(OH)_4_^−^ speciation are illustrated in Fig. 7 and calcification fluid B(OH)_4_^−^/co-precipitating DIC ratios are illustrated in Fig. 6c,d. B(OH)_4_^−^/HCO_3_^−^ and B(OH)_4_^−^/(CO_3_^2−^ + HCO_3_^−^) are positively correlated with pH_CF_ in both the low and high DIC scenarios. As pH_CF_ increases the increase in [B(OH)_4_^−^] (driven by the effect of pH on boron speciation) is larger than any increase in [HCO_3_^−^]_CF_ and [CO_3_^2−^ + HCO_3_^−^]_CF_. In contrast, fluid B(OH)_4_^−^/CO_3_^2−^ is inversely correlated with pH_CF_ at both low and high DIC. Here the proportional increase in [CO_3_^2−^]_CF_ at higher pH_CF_ is larger than any increase in [B(OH)_4_^−^]_CF_. The high DIC scenarios generate lower B(OH)_4_^−^/co-precipitating DIC than the low DIC scenarios at comparable pH because [co-precipitating DIC]_CF_ are higher in the high DIC scenarios. Likewise, [co-precipitating DIC] are higher at 800 μatm seawater pCO_2_ than at 400 μatm seawater pCO_2_ for comparable pH_CF_ yielding lower B(OH)_4_^−^/[co-precipitating DIC] ratios at 800 μatm seawater pCO_2_ (Fig. 6c,d).

### 3.4 Aragonite B/Ca

**Fig. 8:**
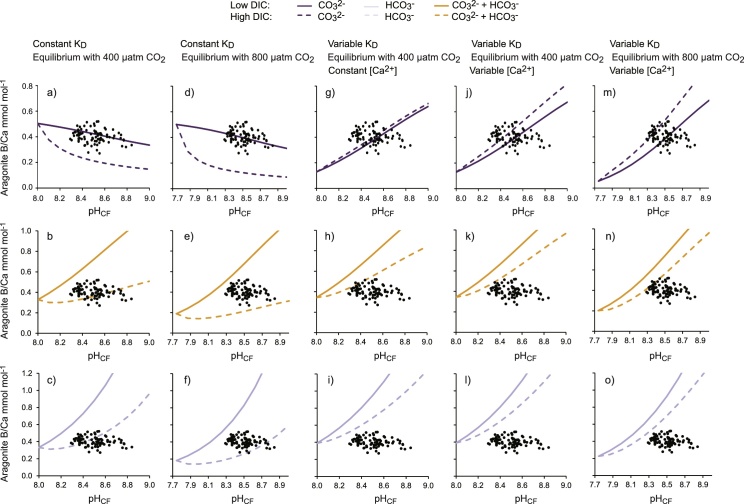
Predicted aragonite B/Ca as a function of pH_CF_ under the different scenarios. Colours indicate different co-precipitating DIC species, solid lines indicate low DIC and dashed lines indicate high DIC. Published relationships between coral calcification pH_CF_ and skeletal B/Ca in corals grown at ambient seawater pCO_2_ are overlain as black dots on each graph.

I calculate the B/Ca of aragonite precipitating from the fluids using the B(OH)_4_^−^/co-precipitating DIC fluid compositions (Fig. 6c,d) and calculated K_D_ (Fig. 5) defined under the different scenarios. I consider a maximum of 48 scenarios (3 co-precipitating scenarios x 2 DIC scenarios x 2CO_2_ atmospheres x 2 [Ca^2+^] scenarios x 2 K_D_ scenarios, Table 1). 30 of these are illustrated in Fig. 8. I do not reproduce the data that combine variable [Ca^2+^]_CF_ (scenario 5a) with a constant K_D_ (scenario 4b) as in setting a constant K_D_ I do not require [Ca^2+^]_CF_ to estimate calcification fluid saturation state. The constant and variable [Ca^2+^]_CF_ scenarios generate broadly similar patterns in skeletal B/Ca. I illustrate the impact of this change at 400 μatm seawater pCO_2_ but calculate expected aragonite B/Ca at 800 μatm seawater pCO_2_ using variable [Ca^2+^]_CF_ only.

#### 3.4.1 Impact of K_D_

Fig. 8a–f indicates aragonite B/Ca assuming that K_D_ does not vary in response to calcification fluid saturation state. Patterns in calcification fluid B(OH)_4_^−^/co-precipitating DIC (Fig. 6c,d) are essentially preserved in aragonite B/Ca. Aragonite B/Ca is inversely correlated with pH_CF_ if B(OH)_4_^−^ co-precipitates with CO_3_^2−^ and is positively correlated with pH_CF_ if B(OH)_4_^−^ co-precipitates with HCO_3_^−^ or (CO_3_^2−^+ HCO_3_^−^). Higher [co-precipitating DIC]_CF_ serves to dilute the calcification fluid B(OH)_4_^−^ and generates lower skeletal B/Ca. For this reason the high DIC scenarios always generate lower skeletal B/Ca compared to the low DIC scenarios at comparable pH_CF_.

Assuming that K_D_ varies in response to calcification fluid saturation state then these patterns change significantly (Fig. 8g–o). K_D_ is positively correlated with fluid saturation state for all co-precipitating DIC species (Fig. 4). In the case that B(OH)_4_^−^ co-precipitates with CO_3_^2−^, [CO_3_^2−^]_CF_ is always relatively high at high pH_CF_ resulting in high K_D_ and enhanced B(OH)_4_^−^ incorporation in the precipitating aragonite. Although fluid B(OH)_4_^−^/[CO_3_^2−^]_CF_ decreases at high pH (Fig. 6c), the increased B(OH)_4_^−^ incorporation at high fluid saturation state overrides this dilution of fluid B(OH)_4_^−^ by CO_3_^2−^. Put simply, over the pH_CF_ range 8 to 9 at 400 μatm seawater pCO_2_, B(OH)_4_^−^/CO_3_^2−^ decreases by x0.7 and x0.3 in the low and high DIC scenarios respectively (Fig. 6c) but K_D_ increases x8 and x22 (assuming a variable [Ca^2+^]_CF_) over the same pH range (Fig. 5b). So in the case that B(OH)_4_^−^ co-precipitates with CO_3_^2−^ and K_D_ is variable, aragonite B/Ca and pH_CF_ are always positively correlated (Fig. 8g,j,m). Higher aragonite B/Ca are generated in the high DIC scenarios than their low DIC counterparts for comparable pH_CF_ because the increase in K_D_ (promoting B(OH)_4_^−^ incorporation in the aragonite) outweighs any B(OH)_4_^−^ dilution by increased fluid [CO_3_^2−^].

Variable K_D_ have a more subtle effect on aragonite B/Ca if B(OH)_4_^−^ co-precipitates with either HCO_3_^−^ only or CO_3_^2−^ + HCO_3_^−^. Assuming that B(OH)_4_^−^ co-precipitates with HCO_3_^−^, then at low DIC both [HCO_3_^−^]_CF_ and K_D_ are relatively high at low pH_CF_ and relatively low at high pH_CF_ (Fig. 6a,b and Fig. 5 respectively). As pH_CF_ increases, the proportional increase in fluid B(OH)_4_^−^/HCO_3_^−^ (Fig. 6c,d) exceeds the proportional decrease in K_D_ and aragonite B/Ca and pH_CF_ are positively correlated (Fig. 8i,l,o). This pattern is maintained at high DIC although in this case the proportional increase in K_D_ (driven by higher [HCO_3_^−^]_CF_ compared to the low DIC scenario) is outweighed by the proportional decrease in fluid B(OH)_4_^−^/HCO_3_^−^ (driven by increased [HCO_3_^−^]_CF_). Thus the high DIC scenarios generate lower aragonite B/Ca than the low DIC scenarios at comparable pH_CF_. Predicted aragonite B/Ca is broadly similar if B(OH)_4_^−^ co-precipitates with both CO_3_^2−^ + HCO_3_^−^ as HCO_3_^−^ is usually the dominant DIC species over the range of pH_CF_. Once again aragonite B/Ca is positively correlated with pH_CF_ in all scenarios and high DIC scenarios generate lower aragonite B/Ca than their low DIC counterparts (Fig. 8h,k,n).

#### 3.4.2 Impact of [Ca^2+^]_CF_

The constant and variable [Ca^2+^]_CF_ scenarios generate broadly similar patterns in skeletal B/Ca (i.e. compare Fig. 8g–i with Fig. 8j–l respectively). [Ca^2+^]_CF_ are higher under the variable [Ca^2+^] scenarios compared to constant [Ca^2+^]_CF_, as proton extrusion by Ca-ATPase serves to increase [Ca^2+^]_CF_. This causes small increases in fluid saturation state and therefore K_D_ (Fig. 5a,b). These higher K_D_ result in higher aragonite B/Ca but the effect is relatively subtle (compare Fig. 8g–i and j–l) as the proportional changes in [Ca^2+^]_CF_ over all scenarios are small compared to changes in [co-precipitating DIC]_CF_.

#### 3.4.3 Impact of seawater pCO_2_

Seawater pCO_2_ has a relatively minor impact on aragonite B/Ca. All [co-precipitating DIC]_CF_ species are increased at 800 μatm seawater pCO_2_ compared to 400 μatm seawater pCO_2_ (Fig. 8). At constant K_D_ these increases dilute the precipitating B(OH)_4_^−^ and decrease aragonite B/Ca. Over all co-precipitation scenarios, changes between predicted aragonite B/Ca at 400 and 800 μatm seawater pCO_2_ are almost imperceptible at low DIC and more significant at high DIC. When K_D_ is variable, the increases in [co-precipitating DIC]_CF_ generate higher K_D_. If B(OH)_4_^−^ co-precipitates with CO_3_^2−^, then the effect of increasing K_D_ outweighs the effect of decreased fluid B(OH)_4_^−^/[CO_3_^2−^] and aragonite B/Ca is higher at 800 μatm compared to 400 μatm seawater pCO_2_. If B(OH)_4_^−^ co-precipitates with HCO_3_^−^ or both CO_3_^2−^ + HCO_3_^−^ then the effect of decreased fluid B(OH)_4_^−^/[co-precipitating DIC] outweighs the effect of increasing K_D_ and aragonite B/Ca is lower at 800 μatm compared to 400 μatm seawater pCO_2_. Under all co-precipitation scenarios at variable K_D_ changes between predicted aragonite B/Ca at 400 and 800 μatm seawater pCO_2_ are very subtle.

#### 3.4.4 Additional factors to consider

**Fig. 9:**
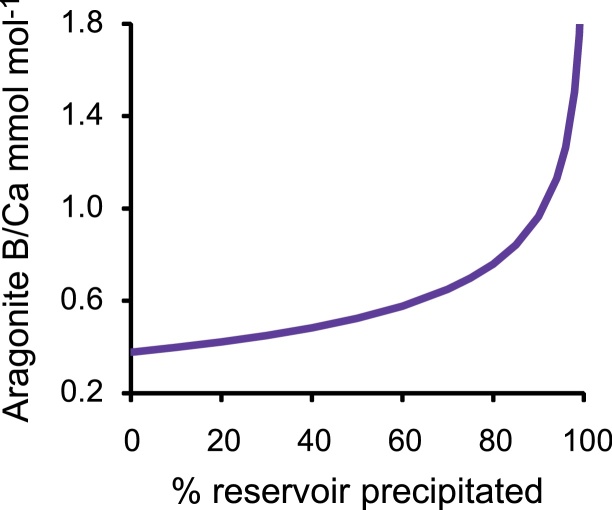
Aragonite B/Ca as a function of the % of the fluid reservoir utilised during precipitation assuming that B(OH)_4_^−^ co-precipitates with CO_3_^2−^. The B(OH)_4_^−^/CO_3_^2−^ aragonite partition coefficient is arbitarily set to 0.00105 when none of the reservoir is utilised.

Rayleigh fractionation can occur when aragonite precipitates from an isolated fluid reservoir (Elderfield et al., 1996). As the K_D_ for all B(OH)_4_^−^/co-precipitating DIC species are much smaller than 1, B(OH)_4_^−^ is preferentially discriminated against during aragonite formation. As precipitation proceeds, the B(OH)_4_^−^/co-precipitating DIC of the fluid remaining in the reservoir, and of the aragonite precipitated from it, increases. The final B/Ca of the precipitated aragonite reflects the proportion of the reservoir used in precipitation (Fig. 9). If Rayleigh fractionation occurs in the coral calcification fluid then skeletal B/Ca will increase when a relatively large proportion of the fluid reservoir is precipitated. This is likely to occur at high fluid saturation states.

Partition coefficients are usually lower in aragonites precipitated on seeds compared to unseeded material (Fig. 4) and the coefficients utilised here may overestimate the calcification fluid DIC of coral aragonite (which precipitates on existing skeletal aragonite).

In the scenario that (B(OH)_4_^−^ co-precipitates with CO_3_^2−^, in some instances the calcification fluid saturation states exceed the maximum state observed in the calculation of K_D_ (Fig. 4). This occurs at pH 9 in the high DIC scenario at 400 μatm seawater pCO_2_ and ≥pH 8.6 in the high DIC scenario at 800 μatm seawater pCO_2_. I have assumed that K_D_ is linearly correlated with calcification fluid saturation state and these high fluid saturation states generate K_D_ (Fig. 5) that exceed the maximum values observed in Fig. 4. Under the growth entrapment model, K_D_ approaches a constant value at high crystal extension rates (assumed to occur at high fluid saturation states) and in this case it is likely that I have overestimated aragonite B/Ca at high pH_CF_ in the high DIC scenario at 800 μatm seawater pCO_2_.

### 3.5 Comparing modelled and observed coral skeletal B/Ca

The observed coral skeletal B/Ca from corals that grew under ambient CO_2_ conditions (i.e. all the data from Fig. 1 with the exception of corals cultured under altered seawater pCO_2_, Honisch et al., 2004) are superimposed onto the graphs in Fig. 8. I do not make any corrections for the different temperatures under which the corals grew. Temperature affects both DIC and boron speciation but the temperature range associated with the data represented in Fig. 1 is small (22–28 °C) and has a relatively minor effect on fluid B(OH)_4_^−^/co-precipitating DIC (<15%). Temperature also has no observable effect on boron partitioning in aragonite above fluid pH of 8.3 (Mavromatis et al., 2015).

Given the assumptions made in the calculations, e.g. in setting [DIC]_CF_ and constant K_D_, I do not attempt to identify a scenario which duplicates the observed coral B/Ca values. However a comparison of the distribution of aragonite B/Ca that can be generated under the different scenarios is informative. Assuming that K_D_ varies in response to fluid saturation state then all co-precipitation scenarios suggest that pH_CF_ and aragonite B/Ca are positively correlated (Fig. 8g–o). Furthermore these scenarios generate relatively narrow ranges of potential B/Ca values (the area of each graph bounded by the low and high DIC scenario lines) irrespective of [DIC] and [Ca^2+^]. At high [co-precipitating DIC] the increase in K_D_ (promoting the incorporation of higher concentrations of B(OH)_4_^−^ in the precipitating aragonite) counteracts the dilution of fluid B(OH)_4_^−^ by high co-precipitating DIC_CF_. In corals the observed skeletal B/Ca is relative constant regardless of inferred pH_CF_ (typically 8.3 to 8.8). This pattern cannot be reproduced by the variable K_D_ scenarios either separately or in combination. I conclude that the coral data cannot be well described by any of the scenarios employing variable K_D_.

The scenarios utilizing constant K_D_ generate wider ranges of aragonite B/Ca that are comparable to the skeletal B/Ca versus pH_CF_ relationships observed in corals (Fig. 8a–f). The observation that coral data is best fitted assuming a constant K_D_ suggests that while coral calcification fluid saturation state may be critical in controlling calcification rate (Gattuso et al., 1999), it is unlikely to affect skeletal extension rate. Under the constant K_D_ scenarios skeletal B/Ca variations are driven by changes in pH_CF_ (affecting boron and DIC speciation) and [DIC]_CF_ (affecting [co-precipitating DIC]_CF_).

Most datasets do not exhibit significant correlations between coral pH_CF_ and skeletal B/Ca (Fig. 1). In the case that B(OH)_4_^−^ co-precipitates with CO_3_^2−^, approximately constant skeletal B/Ca can be generated over a wide pH_CF_ range by a calcification fluid with approximately constant [DIC], as in the low DIC scenario modelled here. Increasing pH_CF_ shifts the DIC equilibrium (Fig. 6a) to increase [CO_3_^2−^]_CF_ which dilutes [B(OH)_4_^−^]_CF_ (Fig. 6c). In the case that B(OH)_4_^−^ co-precipitates with HCO_3_^−^ only or CO_3_^2−^ + HCO_3_^−^ then constant [DIC]_CF_ scenarios generate positive correlations between pH_CF_ and skeletal B/Ca (Fig. 8b,c,e,f) which are inconsistent with the observed coral data. To generate approximately constant skeletal B/Ca over a wide pH_CF_ range under these co-precipitation scenarios requires that [DIC]_CF_ increases at higher pH_CF_. Increasing pH_CF_ serves to increase [DIC]_CF_ probably by facilitating CO_2_ diffusion into the calcification site.

## 4 Conclusions

Observed coral skeletal B/Ca versus pH_CF_ relationships can be reproduced by estimating B(OH)_4_^−^ and co-precipitating DIC speciation as a function of pH_CF_ and assuming that K_D_ are constant i.e. unaffected by calcification fluid saturation state. Assuming that B(OH)_4_^−^ co-precipitates with CO_3_^2−^, then observed patterns can be reproduced by a fluid with approximately constant [DIC] i.e. increasing pH_CF_ concentrates CO_3_^2−^, as a function of DIC speciation. Assuming that B(OH)_4_^−^ co-precipitates with HCO_3_^−^ or CO_3_^2−^ + HCO_3_^−^ then the observed patterns can be reproduced if [DIC]_CF_ and pH_CF_ are positively related i.e. if DIC is increasingly concentrated in the calcification fluid at higher pH_CF_ probably by CO_2_ diffusion into the calcification site.

## Declarations

### Author contribution statement

Nicola Allison: Conceived and designed the experiments; Performed the experiments; Analyzed and interpreted the data; Contributed reagents, materials, analysis tools or data; Wrote the paper.

### Competing interest statement

The authors declare no conflict of interest.

### Funding statement

This work was supported by the UK Natural Environment Research Council (award NE/I022973/1).

### Additional Information

No additional information is available for this paper.
