# Supplementary material for: Reconstructing coral calcification fluid dissolved inorganic carbon chemistry from skeletal boron: An exploration of potential controls on coral aragonite B/Ca
Source: Heliyon. 2017 Aug 30;3(8):e00387. doi: 10.1016/j.heliyon.2017.e00387 (PMC5576960; doi:10.1016/j.heliyon.2017.e00387)
Supplement: Appendix 1 [file mmc1.docx]

| **Appendix 1. Calculation of calcification fluid [DIC] in the high DIC scenarios (see section 2.2.2 and Figure 3).** |  |
| --- | --- |
| 1. Column A - set pH_CF_ | |
| 2. Column B - assume the [DIC] of the initial calcification fluid (derived from seawater) is the same as ambient seawater (1796 and 1911 μmol/kg at seawater pCO_2_ of 400 and 800 μatm respectively) and calculate [CO_2_] using pH_CF_ (column A) and [DIC]. | |
| 3. Column C - assume that the [CO_2_] of the overlying coral tissue is the same as ambient seawater | |
| 4. Column D - calculate ΔC_W_ (the CO_2_ concentration gradient between the initial calcification fluid and the coral tissue) i.e. column C-column B. | |
| 5. Column E - scale the addition of CO_2_ to the calcification fluid by diffusion as a function of ΔC_W_, assuming that CO_2_ diffusion at pH_CF_ 8.5 at 400 μatm seawater pCO_2_ doubles the [DIC] of the final calcification fluid i.e. to 3592 μmol/kg. | |
| 6. Column F - calculate the final [DIC] of the fluid i.e. Column E + 1796 (at 400 μatm seawater pCO_2_) or Column E + 1911 (at 800 μatm seawater pCO_2_) | |
| 7. Assume pH_CF_ is unaffected by CO_2_ invasion i.e. proton extrusion compensates for this. | |
| 8. Use pH_CF_ (column A) and final fluid [DIC] (column F) to calculate concentrations of all DIC species.     \| A \| B \| C \| D \| E \| F \| A \| B \| C \| D \| E \| F \| \| --- \| --- \| --- \| --- \| --- \| --- \| --- \| --- \| --- \| --- \| --- \| --- \| \| pH_CF_ \| Initial [CO_2_]_CF_ \| Tissue [CO_2_] \| ΔCW \| [CO_2_] diffusing in \| Final [DIC]_CF_ \| pH_CF_ \| Initial [CO_2_]_CF_ \| Tissue [CO_2_] \| ΔCW \| [CO_2_] diffusing in \| Final [DIC]_CF_ \| \|  \| μmol/kg \| μmol/kg \| μmol/kg \| μmol/kg \| μmol/kg \|  \| μmol/kg \| μmol/kg \| μmol/kg \| μmol/kg \| μmol/kg \| \| **Seawater pCO_2_ = 400 μatm** \| \| \| \| \| \| **Seawater pCO_2_ = 800 μatm** \| \| \| \| \| \| \| 8.0 \| 11.3 \| 11.3 \| 0.0 \| 0 \| 1796 \| 7.7 \| 22.6 \| 22.6 \| 0.0 \| 0 \| 1911 \| \| 8.1 \| 8.8 \| 11.3 \| 2.5 \| 546 \| 2342 \| 7.9 \| 16.7 \| 22.6 \| 6.0 \| 1283 \| 3194 \| \| 8.2 \| 6.8 \| 11.3 \| 4.5 \| 978 \| 2774 \| 8.0 \| 12.2 \| 22.6 \| 10.4 \| 2245 \| 4156 \| \| 8.3 \| 5.2 \| 11.3 \| 6.1 \| 1319 \| 3115 \| 8.1 \| 8.9 \| 22.6 \| 13.8 \| 2964 \| 4875 \| \| 8.4 \| 3.9 \| 11.3 \| 7.4 \| 1587 \| 3383 \| 8.2 \| 6.4 \| 22.6 \| 16.3 \| 3499 \| 5410 \| \| 8.5 \| 3.0 \| 11.3 \| 8.4 \| 1796 \| 3592 \| 8.4 \| 4.5 \| 22.6 \| 18.1 \| 3895 \| 5806 \| \| 8.6 \| 2.2 \| 11.3 \| 9.1 \| 1959 \| 3755 \| 8.5 \| 3.2 \| 22.6 \| 19.5 \| 4186 \| 6097 \| \| 8.7 \| 1.6 \| 11.3 \| 9.7 \| 2084 \| 3880 \| 8.6 \| 2.2 \| 22.6 \| 20.4 \| 4398 \| 6309 \| \| 8.8 \| 1.2 \| 11.3 \| 10.1 \| 2179 \| 3975 \| 8.8 \| 1.5 \| 22.6 \| 21.2 \| 4550 \| 6461 \| \| 8.9 \| 0.9 \| 11.3 \| 10.5 \| 2251 \| 4047 \| 8.9 \| 1.0 \| 22.6 \| 21.6 \| 4657 \| 6568 \| \| 9.0 \| 0.6 \| 11.3 \| 10.7 \| 2304 \| 4100 \| 9.0 \| 0.6 \| 22.6 \| 22.0 \| 4731 \| 6642 \| | |
